# Short-Term Grazing Exclusion Alters Soil Bacterial Co-occurrence Patterns Rather Than Community Diversity or Composition in Temperate Grasslands

**DOI:** 10.3389/fmicb.2022.824192

**Published:** 2022-03-29

**Authors:** Fangfang Wang, Zongming Li, Bojie Fu, Yihe Lü, Guoping Liu, Dongbo Wang, Xing Wu

**Affiliations:** ^1^State Key Laboratory of Urban and Regional Ecology, Research Center for Eco-Environmental Sciences, Chinese Academy of Sciences, Beijing, China; ^2^College of Resources and Environment, University of Chinese Academy of Sciences, Beijing, China; ^3^Key Laboratory of Agro-Ecological Processes in Subtropical Region and Changsha Research Station for Agricultural & Environmental Monitoring, Institute of Subtropical Agriculture, Chinese Academy of Sciences, Changsha, China; ^4^State Key Laboratory of Earth Surface Processes and Resource Ecology, Faculty of Geographical Science, Beijing Normal University, Beijing, China; ^5^College of Animal Science, Yangtze University, Jingzhou, China; ^6^Hulunbuir Eco-environmental Monitoring Center of Inner Mongolia, Hulunbuir, China

**Keywords:** grazing exclusion, bacterial diversity, bacterial composition, co-occurrence network, temperate grassland

## Abstract

Grazing exclusion is one of the most common practices for degraded grassland restoration worldwide. Soil microorganisms are critical components in soil and play important roles in maintaining grassland ecosystem functions. However, the changes of soil bacterial community characteristics during grazing exclusion for different types of grassland remain unclear. In this study, the soil bacterial community diversity and composition as well as the co-occurrence patterns were investigated and compared between grazing exclusion (4 years) and the paired adjacent grazing sites for three types of temperate grasslands (desert steppe, typical steppe, and meadow steppe) in the Hulunbuir grassland of Inner Mongolia. Our results showed that short-term grazing exclusion decreased the complexity and connectivity of bacterial co-occurrence patterns while increasing the network modules in three types of temperate grasslands. The effects of grazing exclusion on soil bacterial α-diversity and composition were not significant in typical steppe and meadow steppe. However, short-term grazing exclusion significantly altered the community composition in desert steppe, indicating that the soil bacteria communities in desert steppe could respond faster than those in other two types of steppes. In addition, the composition of bacterial community is predominantly affected by soil chemical properties, such as soil total carbon and pH, instead of spatial distance. These results indicated that short-term grazing exclusion altered the soil bacterial co-occurrence patterns rather than community diversity or composition in three types of temperate grasslands. Moreover, our study suggested that soil bacterial co-occurrence patterns were more sensitive to grazing exclusion, and the restoration of soil bacterial community might need a long term (>4 years) in our study area.

## Introduction

Grasslands cover nearly 26% of the global land area and play important roles in providing the base of animal husbandry and promoting human sustainable development (Monfreda et al., 2008; Herrero et al., 2013). In recent decades, due to the intensification of human activities and climate change, grassland degradation has become prevalent worldwide (O’Mara, 2012; Jalaludin et al., 2020). The restoration of degraded grasslands has attracted much attention in recent years. Grazing exclusion is an effective and economical strategy for the restoration of degraded grasslands, which is beneficial for restoring vegetation, improving soil physical structure, and restoring soil nutrients (Su et al., 2005; Tang et al., 2016; Yu et al., 2019). However, previous studies predominantly focused on the effect of grazing exclusion on aboveground biomass and soil physical and chemical properties, while less research has been conducted on the restoration of soil bacterial community.

As the critical component in a grassland ecosystem, soil microorganisms play important roles in maintaining the stability and function of grassland ecosystems, including decomposing the organic matter, driving the elements’ biogeochemical cycle, and regulating the plant nutrient availability (Yao et al., 2011; Zhao et al., 2020). The structure and function of soil microbial community in degraded grasslands is often disintegrated and limited (Zhou et al., 2011). Thus, the reconstruction of soil microbial community is priority in the process of restoration of degraded grasslands. In addition, soil bacterial community can sensitively reflect the change of soil environment (Hawkes and Keitt, 2015). Therefore, the properties of soil bacterial community have been widely used as important biological indexes of soil quality and ecosystem function in the restoration of degraded grassland, especially diversity, composition, and co-occurrence patterns (Zhou et al., 2010; Gao et al., 2021). Network analysis has been widely used to imply co-occurrence patterns in a bacterial community, which can reflect complex community interactions and ecosystem perturbation (Newman, 2006; Zhou et al., 2010).

The temperate grasslands of Inner Mongolia are representative of the Eurasian grassland belt (Wang et al., 2005). In the 1990s, due to the rapid growth of human population and food demand, nearly 50% degradation of the total grassland area was observed in North China (Zhang et al., 2016; Hu and Nacun, 2018), which predominantly resulted from overgrazing. Since 2003, the nationwide conservation project Returning Grazing Lands to Grasslands has been successively implemented in China to restore degraded grasslands. Plenty of studies have discovered that the diversity and biomass of vegetation have been effectively recovered after grazing exclusion in the temperate grasslands of Inner Mongolia (Chen and Tang, 2016). In addition, soil properties, such as organic matter, improved through the decomposition of litter and root exudate, and the soil bulk density decreased after 6–10 years of grazing exclusion (Wu et al., 2014a; Chen and Tang, 2016; Tang et al., 2016). However, the effect of grazing exclusion on the diversity, composition, or co-occurrence patterns of soil bacterial community is inconsistent in previous studies (Cheng et al., 2016; Zhang et al., 2021). In the temperate grassland in Inner Mongolia, meadow steppe, typical steppe, and desert steppe are the three main grassland types with different types of vegetation and an obvious gradient of water availability. The soil bacterial community may differently respond to different types of grasslands. Therefore, it is valuable to make clear the change of soil bacterial community during grazing exclusion in different types of grasslands in Inner Mongolia.

In this study, we compared the differences in the diversity, composition, and co-occurrence patterns of soil bacterial community between short-term grazing exclusion sites and the paired adjacent grazing sites for three types of temperate grasslands (desert steppe, typical steppe, and meadow steppe) in Inner Mongolia. Our objectives are the following: (1) investigate whether short-term grazing exclusion induces the changes in soil bacterial community and (2) investigate whether the response of soil bacterial community to grazing exclusion differs in three types of temperate grasslands.

## Materials and Methods

### Study Area and Soil Sampling

Hulunbuir grassland (47°05′–53°20′ N, 115°31′–126°04′ E), located in the western part of the Greater Khingan Mountains, is a representative temperate grassland in Inner Mongolia and is selected as the research area. The topography is relatively flat, with an altitude of 650–700 m above sea level. The research area has a temperate continental monsoon climate, with mean annual precipitation of 339 mm and mean annual air temperature of −2.2°C from 1980 to 2010. The main soil types in this region are chernozem and kastanozem (Wu et al., 2014b). The dominant plant species in this area are *Aneurolepidium chinense*, *Stipa baicalensis*, and *Carex korshinskyi*.

**FIGURE 1:**
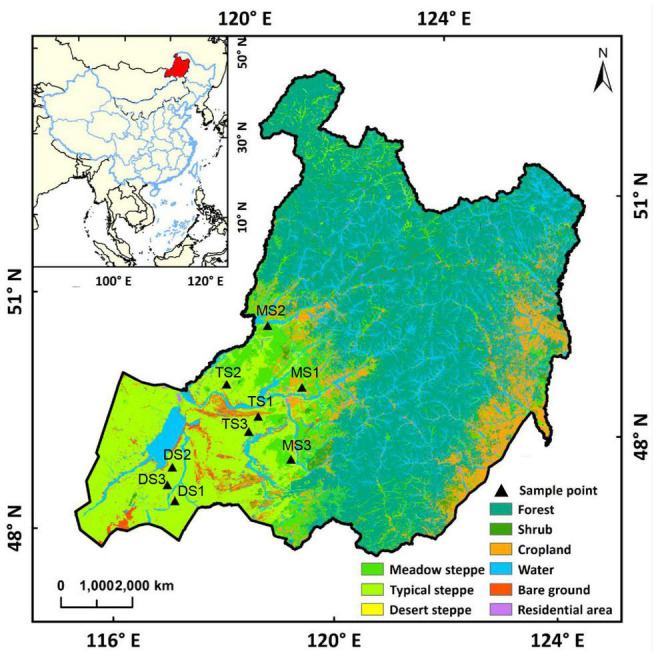
Location of the sampling sites. Each sampling site includes a paired grazing site and grazing exclusion site. MS1, MS2, and MS3 refer to the sampling sites in meadow steppe; TS1, TS2, and TS3 refer to the sampling sites in typical steppe; DS1, DS2, and DS3 refer to the sampling sites in desert steppe.

In July 2019, nine sites covering the main areas of Hulunbuir grassland were selected, that is, three sites in each of the three grassland types: meadow steppe, typical steppe, and desert steppe (Figure 1). At each site, paired plots were sampled, which were a long-term free grazing plot and a nearby grazing exclusion plot (less than 200 m in distance). All the paired plots share the same soil type and similar physiographic conditions, including slope degree, altitude, and topography. Before fencing in 2015, the meadow steppe, typical steppe, and desert steppe sites had been continuously grazed with approximately 9, 7, and 2.7 sheep unit hm^–1^ year^–1^ over the last decades according to local farmers, respectively. The free grazing plots were still grazed when the soil was sampled. The grazing exclusion sites had been fenced without additional management. Besides this, these sites had not been applied with fertilizers. The location, dominant plant species, and main soil characteristics of the sampling sites are shown in Supplementary Table 1. In each plot, four replicate subplots were set (1 m × 1 m) at 10-m intervals along a random transect. The surface (10 cm) soils (the litter layer was removed) were collected by a sterile soil sampler. The soil samples were transported to the laboratory on ice. The sample for extracting microbial DNA was freeze-dried and stored at −80°C. The soil samples for measuring chemical properties were air-dried and stored at 4°C until use.

### Soil Chemical Property Analysis

The soil water content was determined gravimetrically by drying the soil samples at 105°C for 24 h. The soil pH was measured at a soil-to-water ratio of 1:5 using a pH meter (Mettler Toledo, Zurich, Switzerland). The soil total carbon (STC) and soil total nitrogen (STN) contents were determined using an automated C and N analyzer (Elementar, Hanau, Germany). Soil organic carbon (SOC) were determined by potassium dichromate oxidation methods using a spectrophotometer (Lambda25 UV–vis spectrometer, United States). Soil total phosphorus (TP) was measured with Mo–Sb colorimetric method using a spectrophotometer (Lambda25 UV–vis spectrometer, United States). Soil ammonium (NH_4_^+^–N) and nitrate (NO_3_^–^–N) were extracted from 20 g of fresh soil with 1 M KCl (soil/water = 1:5 w/v) and quantified colorimetrically using a flow injection analyzer (Seal AA3, Norderstedt, Germany).

### Bacterial 16S rRNA Gene Sequence Analysis

Soil bacterial community was analyzed using high-throughput sequencing (GeneAmp 9700, ABI, United States). Total microbial DNA was extracted from the soil samples using a FastDNA SPIN Kit for Soil (MP Biochemicals, Solon, OH, United States) following the manufacturer’s instruction. The hypervariable V4 region of bacterial 16S rRNA gene was sequenced by PCR with the primers 515F (5′-GTGCCAGCMGCCGCGG-3′) and 907R (5′-CCGTCAATTCMTTTRAGTTT-3′) (Yusoff et al., 2013). The sequencing was performed on an Illumina MiSeq platform (Illumina, San Diego, CA, United States) at Majorbio Bio-Pharm Technology, Shanghai, China. The obtained raw sequences were processed using Quantitative Insights Into Microbial Ecology (QIIME) with the standard operating procedure (Caporaso et al., 2010; Washburne et al., 2018). Operational taxonomic units (OTUs) were classified at 97% similarity. The representative sequences were then aligned to the Silva database in QIIME2 for maximum sequence similarity.

### Co-occurrence Network of Bacterial Community

The co-occurrence network among bacterial community based on OTU was conducted to investigate the coexistence and interaction patterns of bacteria. The relative abundance of OTUs lower than 0.1% was deleted to reduce the rare OTUs. The significant (*P* < 0.05) and robust correlations (Pearson’s *R* > 0.8) were visualized in the network using Gephi 0.9.2. In the network, the nodes refer to enriched OTUs (relative abundance higher than 0.1%) and edges refer to significant interactive correlations. Four normally used topological properties were calculated in Gephi to describe the complex interaction patterns between OTUs (Newman, 2003). Average degree refers to the average connections of each node with other nodes in the network. Average path length refers to the average distance in shortest paths between two nodes in the network (Faust and Raes, 2012). Average clustering coefficient represents the degree to which the nodes tend to cluster together. Modularity class quantifies the extent to which the network can be divided into different clusters (Rottjers and Faust, 2018). Modularity value >0.4 suggests that the network has a modular structure, and the nodes are highly connected within the cluster but less connected outside the cluster (Newman, 2006). Module in the network is densely connected clusters of nodes and has been interpreted as ecological niche preferences (Chaffron et al., 2010; Freilich et al., 2010). Average degree and average path length are used to measure the connectivity of the network. Average clustering coefficient and modularity can reflect the cohesion of the network.

### Statistical Analysis

All data are presented as mean and standard error. Pearson correlations were performed in SPSS 21 (IBM, Armonk, NY, United States). Statistically significant differences were accepted when *P* < 0.05. Scatter diagram and bar plot were generated in OriginPro 2018 (Origin Lab Corporation, United States). The β-diversity of the bacterial community was estimated based on Bray–Curtis distances in “vegan” package and plotted using “ggplot2” package in R 3.4.3. ANOSIM was performed to test the significance in difference of community composition between groups in “vegan” package in R 3.4.3 (Oksanen et al., 2020). RDA was conducted in “vegan” package in R 3.4.3.

## Results

### Effect of Grazing Exclusion on Soil Properties

Before grazing exclusion, soil total carbon was significantly higher in meadow steppe than typical steppe and desert steppe soils, while the difference between typical steppe and desert steppe was not significant (Table 1). The difference of other soil chemical properties among three grassland types was not significant. After short-term grazing exclusion, the TP of desert steppe significantly decreased while STC and STN were slightly decreased. In typical steppe, grazing exclusion significantly increased the STC by 13%. TP, NH_4_^+^–N, and NO_3_^–^–N were slightly decreased. In meadow steppe, grazing exclusion induced a minor increase of the determined soil chemical properties, but the change was not significant. Short-term grazing exclusion increased the difference of STN and TP among the three grassland types.

**TABLE 1:** Soil chemical properties between grazing and grazing exclusion treatments.

Grassland type	STC (g C kg^–1^)		STN (g N kg^–1^)		SOC (g C kg^–1^)		TP (g P kg^–1^)		NH_4_^+^–N (mg N kg^–1^)		NO_3_^–^–N (mg N kg^–1^)	
	Grazing	Exclusion	Grazing	Exclusion	Grazing	Exclusion	Grazing	Exclusion	Grazing	Exclusion	Grazing	Exclusion
Desert steppe	22.3 ± 1.4 Ba	21.2 ± 1.9 Ba	2.1 ± 0.3 Aa	1.9 ± 0.3 Ba	19.6 ± 0.7 Ba	20.4 ± 2.2 Ba	0.50 ± 0.06 Aa	0.38 ± 0.03 Bb	12.1 ± 0.8 Aa	12.2 ± 0.2 Aa	8.1 ± 1.7 Ab	18.8 ± 6.1 Aa
Typical steppe	25.6 ± 6.6 Bb	29.1 ± 7.1 Ba	2.2 ± 0.5 Aa	2.5 ± 0.5 Ba	23.5 ± 5.6 Bb	25.9 ± 5.6 Ba	0.37 ± 0.08 Aa	0.35 ± 0.05 Ba	14.6 ± 3.4 Aa	12.6 ± 1.7 Aa	10.2 ± 4.0 Aa	6.4 ± 1.3 Aa
Meadow steppe	34.9 ± 4.4 Aa	38.6 ± 4.1 Aa	2.7 ± 0.1 Aa	3.2 ± 0.3 Aa	31.0 ± 4.6 Aa	33.9 ± 3.2 Aa	0.40 ± 0.02 Aa	0.45 ± 0.02 Aa	13.7 ± 2.3 Aa	13.4 ± 1.6 Aa	11.4 ± 8.8 Aa	14.2 ± 11.1 Aa

*The means (± SD, N = 4) for each variable followed by different lowercase letters indicate significant differences between grazing and exclusion sites (P < 0.05, two-tailed paired t-test). The uppercase letters indicate significant difference among different grassland types (P < 0.05, one-way ANOVA, least significant difference). STC, soil total carbon; STN, soil total nitrogen; SOC, soil organic carbon; TP, total phosphorus.*

### Effect of Grazing Exclusion on Soil Bacterial Community Diversity and Composition

**FIGURE 2:**
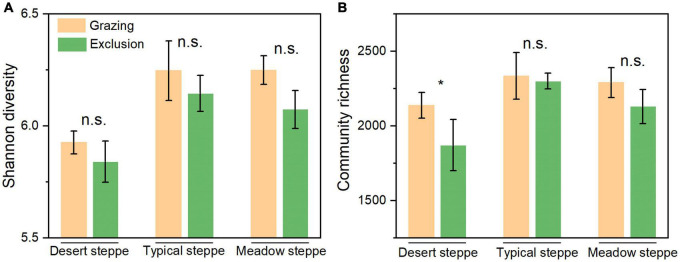
The bacterial α-diversity **(A)** based on Shannon index and community richness **(B)** based on Sobs index between grazing and exclusion sites in three types of grasslands. Error bars indicate standard errors (3 replicate sites). “*” means significant difference (*P* < 0.05) between grazing and exclusion sites. “n.s.” means non-significant.

**FIGURE 3:**
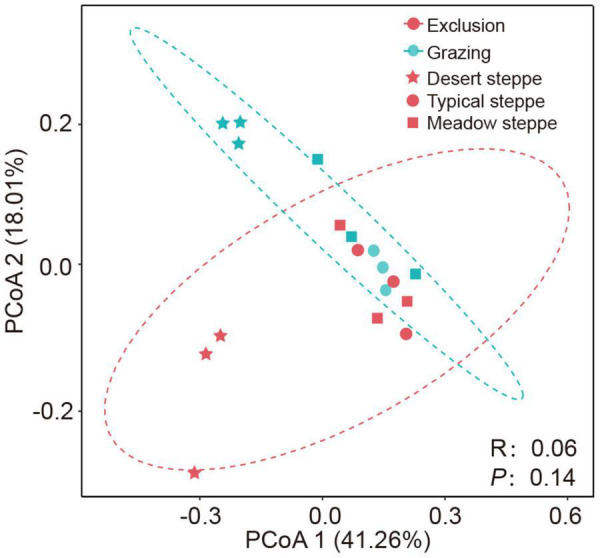
Bacterial community structure assessed by β-diversity patterns using the principal coordinate analysis plots of Bray–Curtis distances. Different color represents exclusion or grazing soils and shape represents grassland types: desert steppe, typical steppe, and meadow steppe. ANOSIM similarity analysis was used to test the significance between groups.

At the phylum level, Actinobacteria, Proteobacteria, Actinobacteria, and Chloroflexi are the dominant bacteria, which account for 44, 15, 13, and 10% of the total bacteria, respectively (Supplementary Figure 1). In grazing sites, only the relative abundance of Proteobacteria was significantly higher in typical steppe than in desert steppe and meadow steppe (*P* < 0.01), and the difference in the relative abundance of the other major phyla was not significant (Supplementary Table 2). However, after short-term grazing exclusion, the difference of the relative abundance of bacterial phyla between exclusion and grazing sites was not significant in the three types of grasslands. The α-diversity of the bacterial community based on Shannon index is significantly lower in desert steppe than in typical steppe and meadow steppe in grazing sites (*P* = 0.018, Supplementary Table 2). The difference of community richness based on Sobs index was not significant among the three types of grasslands. After grazing exclusion, α-diversity was decreased in the exclusion treatments, but the difference was not significant (Figure 2A). The community richness in the exclusion treatments was significantly lower than in the grazing treatments in desert steppe (*P* < 0.05, Figure 2B). However, the difference of community richness between exclusion and grazing was not significant in typical steppe and meadow steppe. The bacterial β-diversity of Bray–Curtis distance among the three grassland types was significant based on ANOSIM analysis (*P* < 0.01). However, the β-diversity between exclusion and grazing treatments was not significant (*P* = 0.14, Figure 3).

### Effect of Grazing Exclusion on Soil Bacterial Co-occurrence Patterns

**FIGURE 4:**
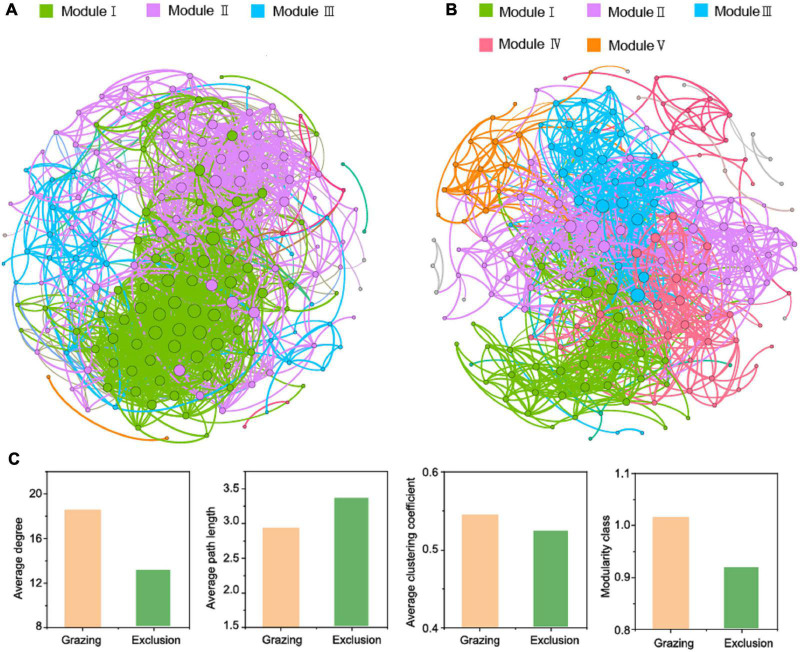
Network analysis depicts the co-occurrence patterns among bacterial community based on operational taxonomic unit (Pearson’s *R* > 0.8, *P* < 0.05) for grazing **(A)** and exclusion **(B)** treatments as well as the topological indexes **(C)**. The node size indicates connectivity degree. The colors of the nodes and edges are grouped by modularity class. Different colors refer to different modules.

The co-occurrence networks were established to investigate bacterial co-existence and interaction for the grazing and exclusion treatments. As shown in Figures 4A,B, 184 and 201 enriched OTUs formed 1,413 and 1,465 significant and robust associations in the grazing and exclusion treatments, respectively. The topological properties of the network analysis were calculated (Figure 4C). Compared with the exclusion treatments, the grazing treatments exhibited higher average degree, higher average clustering coefficient, and lower average path length, indicating that the grazing treatments have higher connectivity and closer connection in the network. The modularity classes are both higher than 0.4 in the grazing and exclusion treatments, which suggested that the enriched OTUs both exhibited a highly modular structure. In addition, the co-occurrence network in the grazing exclusion treatments exhibited five network modules, while only three network modules were found in the grazing treatments.

### Relationships Between Soil Properties and Bacterial Community

**FIGURE 5:**
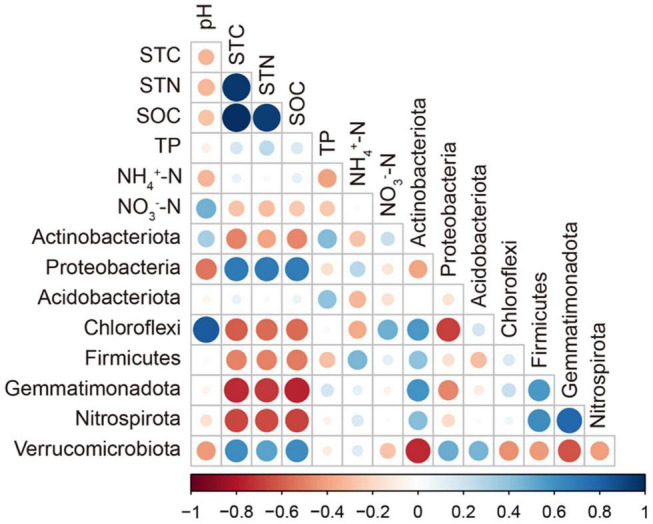
Correlation between soil properties and the major bacterial phyla. Circle size and color represent the Pearson correlation coefficient.

**FIGURE 6:**
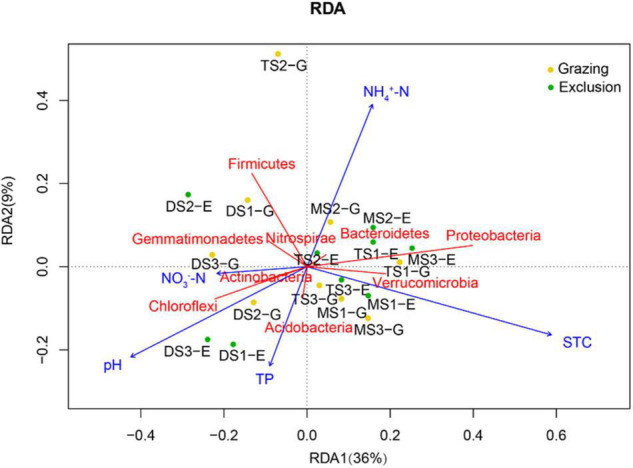
Redundancy analysis of the significant soil chemical properties on the composition of bacterial community on phylum level. Blue arrows refer to soil chemical properties, red solid lines refer to bacterial phyla, and yellow and green dots refer to sampling sites.

The correlations between main soil chemical properties and bacterial α-diversity were not significant in the three types of grasslands of grazing exclusion and grazing soils (Supplementary Table 3). The correlations between soil properties and major bacterial phyla are shown in Figure 5. The relative abundance of Proteobacteria and *Verrucomicrobia* was significantly positively correlated with the content of STC, STN, and SOC. The relative abundance of Actinobacteria, Choloflexi, Firmicutes, Gemmatimonadota, and Nitrospirota was significantly negatively correlated with STC, STN, and SOC. The pH was significantly correlated with the relative abundance of Chloroflexi. In addition, RDA was conducted to investigate the effect of soil chemical properties on the composition of the bacterial community (Figure 6). The adjusted *R*^2^ of the RDA model was 0.498, which means that 49.8% of the total variance of the bacterial community can be explained by the selected soil chemical properties in the grazing and exclusion soils. Among them, STC played a leading role in affecting the distribution of the bacterial community, followed by pH, NH_4_^+^–N, TP, and NO_3_^–^–N.

## Discussion

### Effect of Grazing Exclusion on Bacterial Diversity and Composition

In our study, short-term grazing exclusion decreased the α-diversity in the three types of temperate grasslands, but the differences were not significant between the exclusion and grazing treatments. In addition, grazing exclusion did not induce significant changes of the composition and the relative abundance of dominant phyla in typical steppe and meadow steppe. A previous study reported that 7 years of grazing exclusion of upland grassland decreased the soil bacterial diversity as a result of inhibition of soil carbon and nitrogen cycling (Medina-Roldan et al., 2012). Another study also found that the composition of soil bacterial communities was not influenced by 6 years of restoration in a tall-grass prairie in northeastern Kansas (Murphy and Foster, 2014). These results are consistent with our findings. However, other studies had found different results—for example, Yao et al. (2018) found that the composition of soil bacterial community significantly changed after 13 years of fencing in *Leymus chinensis* and *Stipa grandis* steppe. One possible explanation for the different effect of grazing exclusion on community properties is the exclusion duration. Short-term grazing exclusion might not induce significant changes of bacterial diversity and composition in typical steppe and meadow steppe. This may be because the response of soil bacterial community is slow and unresponsive to short-term grazing exclusion (Nunan et al., 2005; Singh et al., 2007). Therefore, the change of soil bacterial diversity and composition through short-term grazing exclusion is not evident or may take a long duration. Grazing exclusion significantly reduced community richness and changed community composition only in desert steppe, which may be attributed to different grazing history and soil property (Huhe et al., 2017; Yao et al., 2018).

### Effect of Grazing Exclusion on Soil Bacterial Co-occurrence Patterns

The co-occurrence networks offer insight into the complex interactions between bacteria. These also reflect the associations between bacterial community and environment. Firstly, the co-occurrence patterns of bacterial community can be used as good indicators of grassland ecosystem perturbation (Zhou et al., 2010). As shown in the co-occurrence networks, the bacterial connectivity and interactions are closer and more complex in grazing treatments than in exclusion treatments. The reason may be that grazing soils experience more disturbances from livestock, thus forming more complex linkages to face the challenge of limited resources (Zhang et al., 2018). Although the total amount of soil carbon and nitrogen content did not differ significantly between the grazing and grazing exclusion treatments, the availability of soil carbon and nitrogen content may decrease due to the deterioration of the soil physical conditions in grazing soils (Wang et al., 2021). Therefore, without livestock disturbances, the complexity of a bacterial co-occurrence network decreased in grazing exclusion treatments. A higher value of average path length (AL) has been interpreted to decrease the speed of a bacterial network’s response to perturbations (Zhou et al., 2010), which is consistent with the higher AL of the exclusion treatments than the grazing treatments. Secondly, the module is interpreted as a similar environment where microbes share overlapping ecological niches (Barberan et al., 2012). High modularity indicates that the boundary is clear between different modules in the network, and it is also considered as having highly distinguished niches in the community (Chaffron et al., 2010; Freilich et al., 2010; Faust and Raes, 2012), which is exhibited in both grazing and grazing exclusion treatments. Moreover, exclusion soils exhibited more diverse modules than grazing soils, which may be attributed to abundant plant-derived resources and improved soil environments creating more diverse niches for microorganisms (Chen et al., 2020; Lin et al., 2021)—for example, the increased plant root exudates and little decomposition with accumulation of aboveground plant biomass (Cheng et al., 2016). In the same ecological niche, microbes which are sharing resource may form more diverse interactions, including cooperation or competition (Freilich et al., 2010). Therefore, more diverse modules in the grazing exclusion treatments indicate more diverse interactions. In conclusion, short-term grazing exclusion altered the co-occurrence patterns of soil bacterial community, especially increasing the diversity of interactions in the three types of temperate grasslands.

### Soil Properties That Affect Bacterial Community Composition

Whether grazing exclusion can change the diversity and composition of soil bacterial community or not, soil chemical properties are the deterministic factors (Zhang et al., 2018; Li et al., 2019; Qin et al., 2021). As shown in RDA, the main soil chemical properties, especially pH, STC, and TP, are the dominant factors that affect the dissimilarity of soil bacterial community. Previous studies also found that pH (Lauber et al., 2009) and the availability of carbon and nitrogen (Sul et al., 2013; Cederlund et al., 2014) have predominant influences on the abundance and structure of soil microbial community in varied scales. Several mechanisms may explain the deterministic effect of edaphic factors on soil bacterial community. Firstly, pH can directly pose selective pressures on bacteria, as specific species poses different tolerance ability to pH—for example, our study found that the relative abundance of Choloflexi was positively correlated with pH, while the relative abundance of Proteobacteria and Verrucomicrobia showed negative correlations. pH can also indirectly affect the composition of bacterial community as it is normally related with multiple soil factors, including nutrient availability, redox state, and salinity (Lauber et al., 2009; Ren et al., 2020). Secondly, available carbon and nitrogen are the two key resources supporting the survival of most terrestrial heterotrophic microorganisms. Plenty of studies have found that the quality and the quantity of carbon and nitrogen determine the structure of soil bacterial community (Murphy and Foster, 2014; Zhang et al., 2018). Furthermore, soil chemical properties also showed close correlations with some members of the bacterial community. Our study found that the content of carbon and nitrogen exhibited positive correlations with Proteobacteria and Verrucomicrobiota, which is consistent with a previous study indicating that most types of the two phyla are heterotrophic microorganisms (Padhy et al., 2021). In addition, in our study, short-term grazing exclusion did not induce significant changes of the main soil chemical properties, especially pH, STC, and STN, which may explain that the response of bacterial community was not significant.

## Conclusion

Our results showed that short-term grazing exclusion did not induce significant changes of the diversity and composition of soil bacterial community in typical steppe and meadow steppe but altered the community composition in desert steppe. The complexity and connectivity of bacterial co-occurrence patterns decreased, while the diversity of interactions increased through grazing exclusion management among the three types of temperate grasslands. In addition, soil chemical properties, especially pH, STC, and TP, are the dominant factors that affect the composition of soil bacterial community. These results indicated that the effect of grazing exclusion differs in three main grassland types in Inner Mongolia. Moreover, soil bacterial co-occurrence interactions may be more sensitive to grazing exclusion, and the restoration of soil bacterial community in our study area might need a long term (>4 years).

## Data Availability Statement

The 16S rRNA gene sequencing data of all samples were submitted to the NCBI SRA database (https://www.ncbi.nlm.nih.gov/) under accession number PRJNA787389.

## Author Contributions

FW and XW conceptualized this study and led the writing. ZL, GL, and DW collected and analyzed the data. BF, YL, and XW interpreted the results and revised the text. All authors contributed to this work and approved the final manuscript before submission.

## Conflict of Interest

The authors declare that the research was conducted in the absence of any commercial or financial relationships that could be construed as a potential conflict of interest.

## Publisher’s Note

All claims expressed in this article are solely those of the authors and do not necessarily represent those of their affiliated organizations, or those of the publisher, the editors and the reviewers. Any product that may be evaluated in this article, or claim that may be made by its manufacturer, is not guaranteed or endorsed by the publisher.
