# Supplementary material for: Short-Term Grazing Exclusion Alters Soil Bacterial Co-occurrence Patterns Rather Than Community Diversity or Composition in Temperate Grasslands
Source: Front Microbiol. 2022 Mar 29;13:824192. doi: 10.3389/fmicb.2022.824192 (PMC9005194; doi:10.3389/fmicb.2022.824192)
Supplement: Supplementary file 1 [file Data_Sheet_1.docx]

Supplementary Figure 1 Stacked bar chart showing the relative abundance of bacteria at phylum level. Values are the average relative abundance of 3 samples in grazing or exclusion sites in the same grassland type; error bars indicate standard errors (N = 3).





Supplementary Table 1 The location and main characteristics of the sampling sites. Values are the mean of 4 replicate plots (±SD).

| Sampling sites | Location | Grassland type | Treatment | Dominant species | pH | STC  (g C kg^-1^) | STN  (g N kg^-1^) | SOC  (g C kg^-1^) | TP  (g P kg^-1^) | NH_4_^+^-N  (mg N kg^-1^) | NO_3_^-^-N  (mg N kg^-1^) |
| --- | --- | --- | --- | --- | --- | --- | --- | --- | --- | --- | --- |
| DS1-E | 48.14 N, 117.40 E | Desert steppe | Exclusion | *Allium polyrrhizum,*  *Carex korshinskii* | 7.81±0.03 | 19.00±1.67 | 1.66±0.23 | 18.17±0.38 | 0.35±0.01 | 12.08±0.56 | 23.05±1.29 |
| DS1-G | 48.14 N, 117.40 E | Desert steppe | Grazing | *Carex korshinskii,*  *Allium polyrhizum* | 7.25±0.02 | 21.47±1.09 | 1.86±0.74 | 18.87±1.20 | 0.45±0.04 | 12.16±1.62 | 7.46±1.50 |
| DS2-E | 48.35 N, 117.29 E | Desert steppe | Exclusion | *Allium polyrrhizum,*  *Carex korshinskii* | 7.83±0.03 | 21.98±1.45 | 2.08±0.52 | 20.27±1.10 | 0.38±0.02 | 12.03±0.97 | 21.37±2.26 |
| DS2-G | 48.35 N, 117.29 E | Desert steppe | Grazing | *Carex korshinskii,*  *Allium polyrhizum* | 7.18±0.13 | 23.88±1.15 | 2.37±0.19 | 20.21±0.78 | 0.51±0.03 | 12.85±0.79 | 10.11±2.58 |
| DS3-E | 48.57 N, 117.42 E | Desert steppe | Exclusion | *Allium polyrrhizum,*  *Carex korshinskii* | 7.78±0.01 | 22.60±1.05 | 2.09±0.23 | 22.62±2.19 | 0.41±0.02 | 12.40±1.31 | 11.83±1.35 |
| DS3-G | 48.57 N, 117.42 E | Desert steppe | Grazing | *Carex korshinskii,*  *Allium polyrhizum* | 7.24±0.02 | 21.52±1.36 | 2.03±0.23 | 19.62±0.48 | 0.56±0.03 | 11.29±0.23 | 6.85±2.64 |
| TS1-E | 49.06 N, 119.16 E | Typical steppe | Exclusion | *Aneurolepidium chinense ,*  *Cleistogenes squarrosa* | 7.27±0.05 | 35.51±1.86 | 2.88±0.31 | 30.88±2.01 | 0.35±0.02 | 13.52±1.23 | 7.8±0.62 |
| TS1-G | 49.06 N, 119.16 E | Typical steppe | Grazing | *Carex korshinskii* | 7.19±0.03 | 31.96±2.34 | 2.72±0.20 | 28.38±1.51 | 0.38±0.01 | 12.61±0.99 | 9.93±2.27 |
| TS2-E | 49.52 N, 118.65 E | Typical steppe | Exclusion | *Aneurolepidium chinense,*  *Cleistogenes squarrosa* | 7.14±0.21 | 21.48±1.09 | 1.92±0.16 | 19.84±1.21 | 0.31±0.01 | 13.54±0.53 | 6.36±1.49 |
| TS2-G | 49.52 N, 118.65 E | Typical steppe | Grazing | *Carex korshinskii* | 7.09±0.15 | 18.75±0.53 | 1.69±0.07 | 17.41±0.85 | 0.29±0.05 | 18.62±1.21 | 14.38±2.04 |
| TS3-E | 48.89 N, 118.95 E | Typical steppe | Exclusion | *Aneurolepidium chinense,*  *Cleistogenes squarrosa* | 7.07±0.05 | 30.24±2.17 | 2.66±0.10 | 26.86±2.37 | 0.4±0.02 | 10.58±0.43 | 5.13±0.79 |
| TS3-G | 48.89 N, 118.95 E | Typical steppe | Grazing | *Carex korshinskii* | 7.27±0.07 | 26.04±1.15 | 2.23±0.12 | 24.7±0.92 | 0.45±0.01 | 12.59±0.97 | 6.33±0.61 |
| MS1-E | 48.47 N, 119.66 E | Meadow steppe | Exclusion | *Carex korshinskii,*  *Stipa baicalensis* | 7.37±0.02 | 38.91±1.86 | 3.17±0.18 | 33.72±1.20 | 0.46±0.03 | 11.51±0.23 | 12.37±0.32 |
| MS1-G | 48.47 N, 119.66 E | Meadow steppe | Grazing | *Carex korshinskii,*  *Aneurolepidium chinense* | 7.25±0.03 | 32.11±0.77 | 2.66±0.10 | 27.75±1.66 | 0.38±0.01 | 11.43±1.10 | 6.89±1.65 |
| MS2-E | 50.19 N, 119.60 E | Meadow steppe | Exclusion | *Carex korshinskii,*  *Cleistogenes squarrosa* | 7.03±0.05 | 34.36±1.45 | 2.89±0.13 | 30.82±1.64 | 0.43±0.01 | 14.3±0.71 | 26.11±2.16 |
| MS2-G | 50.19 N, 119.60 E | Meadow steppe | Grazing | *Carex korshinskii,*  *Aneurolepidium chinense* | 7.14±0.04 | 32.84±2.09 | 2.7±0.20 | 28.93±1.71 | 0.4±0.01 | 13.58±0.53 | 21.49±2.27 |
| MS3-E | 49.35 N, 120.07 E | Meadow steppe | Exclusion | *Aneurolepidium chinense,*  *Carex korshinskii* | 7.29±0.02 | 42.58±1.18 | 3.48±0.10 | 37.27±1.15 | 0.46±0.02 | 14.36±2.92 | 4.08±1.67 |
| MS3-G | 49.35 N, 120.07 E | Meadow steppe | Grazing | *Carex korshinskii,*  *Aneurolepidium chinense* | 7.16±0.02 | 39.98±2.74 | 2.8±0.10 | 36.19±2.73 | 0.42±0.04 | 16.04±1.82 | 5.84±0.97 |

Notes: Sampling sites with the same prefix are paired sites, such as DS1-E and DS1-G. STC: soil total carbon. STN: soil total nitrogen. SOC: soil organic carbon. TP: total phosphorus.

Supplementary Table 2 The differences of bacterial Shannon and sobs index, the abundance of major phyla among three types of grasslands in grazing sites.

| Grassland type | Shannon index | Sobs index | Actinobacteria | Proteobacteria | Acidobacteria | Chloroflexi | Firmicutes | Gemmatimonadetes | Nitrospirae | Verrucomicrobia |
| --- | --- | --- | --- | --- | --- | --- | --- | --- | --- | --- |
| Desert steppe | 5.93±0.06 b | 2138.33±105.56 a | 15751±2504.46 a | 2962±414.82 b | 4944±1644.18 a | 3399±731.9 a | 1416±608.73 a | 1471±153.03 a | 168±30.89 a | 466±196.17 a |
| Typical steppe | 6.25±0.16 a | 2334.67±190.43 a | 14150±1524.06 a | 7184±1679.35 a | 4127±1447.39 a | 2680±237.39 a | 1929±2241.42 a | 1216±467.87 a | 215±58.66 a | 826±786.63 a |
| Meadow steppe | 6.25±0.08 a | 2290.33±123.22 a | 12950±1373.91 a | 5825±242.59 a | 5122±1298.75 a | 2705±219.49 a | 750±504.82 a | 759±142.31 a | 115±43.41 a | 1290±486.61 a |

Notes: Means (±SD, 3 replicate sites) for each variable followed by different letters indicate significant differences among three types of grasslands (P < 0.05, One-way ANOVA, LSD).

Supplementary Table 3 Pearson correlation between bacterial Shannon and sobs index, the abundance of major phyla and soil chemical properties.

|  | pH | STC  (g C kg-1) | STN  (g N kg-1) | SOC  (g C kg^-1^) | TP  (g P kg-1) | NH_4_^+^-N  (mg N kg^-1^) | NO_3_^-^-N  (mg N kg^-1^) |
| --- | --- | --- | --- | --- | --- | --- | --- |
| Shannon index | -0.51* | 0.33 | 0.28 | 0.32 | -0.53* | 0.14 | -0.14 |
| sobs index | -0.71** | 0.25 | 0.22 | 0.19 | -0.24 | 0.15 | -0.19 |
| Actinobacteria | 0.35 | -0.45 | -0.35 | -0.44 | 0.40 | -0.29 | 0.22 |
| Proteobacteria | -0.55* | 0.70** | 0.69** | 0.69** | -0.21 | 0.29 | -0.14 |
| Acidobacteria | -0.04 | 0.08 | 0.03 | 0.07 | 0.40 | -0.35 | -0.16 |
| Chloroflexi | 0.84** | -0.60** | -0.56* | -0.56* | 0 | -0.37 | 0.50* |
| Firmicutes | 0.02 | -0.50* | -0.49* | -0.51* | -0.29 | 0.45 | 0.11 |
| Gemmatimonadetes | -0.06 | -0.74** | -0.70** | -0.75** | 0.19 | 0.19 | -0.08 |
| Nitrospirae | -0.13 | -0.67** | -.66** | -0.67** | -0.09 | 0.16 | 0.05 |
| Verrucomicrobia | -0.42 | 0.63** | 0.53* | 0.62** | -0.09 | 0.14 | -0.28 |

Notes: Values are Pearson correlation coefficient. *: *P* < 0.05, **: *P* < 0.01. STC: soil total carbon. STN: soil total nitrogen. SOC: soil organic carbon. TP: total phosphorus.
